# Community perceptions of natural supports and their approaches: A Canadian grounded theory study with Connections First

**DOI:** 10.1371/journal.pone.0346971

**Published:** 2026-05-04

**Authors:** Jessica-Lynn A. Walsh, Kiran Pohar Manhas, Nikki Stephenson, Sheila W. McDonald, Scott B. Patten, Suzanne C. Tough

**Affiliations:** 1 Department of Pediatrics, Cumming School of Medicine, University of Calgary, Calgary, Alberta, Canada; 2 Department of Medical Sciences, Cumming School of Medicine, University of Calgary, Calgary, Alberta, Canada; 3 Neurosciences, Rehabilitation and Vision, Strategic Clinical Network, Alberta Health Services, Calgary, Alberta, Canada; 4 Department of Obstetrics and Gynecology, Cumming School of Medicine, University of Calgary, Calgary, Alberta, Canada; 5 Department of Community Health Sciences, Cumming School of Medicine, University of Calgary, Calgary, Alberta, Canada; 6 Cuthbertson & Fischer Chair in Pediatric Mental Health, Department of Psychiatry, Cumming School of Medicine, University of Calgary, Calgary, Alberta, Canada; Oregon State University, UNITED STATES OF AMERICA

## Abstract

Community-based informal reciprocal interactions (natural supports) enhance individual and community well-being. Like social connections, natural supports aim to establish supportive and healthy environments, but with a greater focus on local surroundings. This study explored natural support approaches within communities in an urban Canadian center. Employing grounded theory, purposive and theoretical sampling identified participants familiar with the community and its opportunities and activities, referred to as community champions. These champions were interviewed about their perceptions of resident connectivity and the key facilitators and barriers related to natural supports approaches in their communities. Themes and categories emerged, leading to the development of a theory. The overarching theory posited “*building a community’s capacity to transition from disconnected to naturally supportive: the need for connectors, assets, and action to empower residents”.* Community connectors and assets facilitate natural support approaches within urban community settings. Limited access to space and challenges in recruiting and retaining volunteers were identified as barriers. The findings empower knowledge users, such as community planners, to invest in and promote community natural supports approaches to enhance resident and community well-being. Future directions for this study include the implementation and evaluation of natural support approaches within communities.

## Introduction

It is well-established that social connections and supportive environments promote positive physical, psychological, and developmental outcomes [1–5]. However, research shows that social connections, social participation (engagement in community or societal opportunities) [6], and social engagement (active participation in social activities, roles, and norms) [7] have all declined over the past several decades [6,8]. For example, the Human Early Learning Partnership collected data from Grade 4 students in 19 British Columbia (BC) school districts in Canada; they observed an increase in the number of children self-reporting that they have “no important adults” in their lives from 15% in 2017–2018 to 29% in 2018–2019 [9]. In adult populations, US national trends between 2003 and 2020 found that social isolation (an objective measure of a lack of social connections, interactions, or memberships) [6] among individuals aged 15 years and older has increased [10]. There is a recognized need to promote innovative and sustainable strategies that foster social connection [11]. Our study presents an opportunity to utilize informal, natural support approaches within community settings to foster social connections and supportive environments, drawing on an evidence-based theory that can guide these approaches.

### One potential mechanism: Natural supports

Natural supports have emerged from academic, non-profit, and government literature and reports within community contexts [12–14]. Natural supports are informal reciprocal connections that consist of close relationships with friends and family, as well as broader associations, including neighbours, organizations, and local businesses [14]. Similar to social connections, natural supports aim to create supportive and healthy environments, while also emphasizing the locality or places where individuals and families spend their time [14]. Although the concept of natural supports is not new, particularly in disability literature, it is a relatively recent idea in health and community literature. This study arose from a need for a sustainable approach to fostering a supportive social environment beyond professional and formal supports and services [15]. For the purposes of this study, we focused on natural supports involving informal connections among residents, organizations, local businesses, and other community-based entities.

In 2017, the Change Collective, a consortium of non-profit organizations in Alberta, Canada, released a Practice Framework for organizations and health service providers to improve non-professional support for vulnerable youth, particularly those transitioning into adulthood [14]. However, there is a lack of peer-reviewed literature on community-based natural supports, and no theories adequately capture how to promote informal natural supports in community settings.

### Rationale and objective

Organizations and governments have begun to prioritize incorporating communities into their care models and supporting community-based strategies aimed at strengthening resident and family health and well-being [16–18]. Formal community-based programming and interventions, such as social prescription, are becoming increasingly common to promote social connections that support positive health outcomes [19]. Social prescription involves linking patients in primary care to supports and resources available within community settings [20]. However, there is a lack of evidence regarding the perceptions of barriers and facilitators related to developing *informal* approaches that foster supportive connections and environments within communities for residents and families; thus, exploring natural supports becomes an ideal strategy for addressing this gap. Understanding natural supports approaches from the perspectives of individuals working and living in community settings is crucial for advancing and implementing informal strategies that empower communities to support residents in promoting positive health and well-being [21–25]. Therefore, the objective of this study was to investigate community residents’ and leaders’ perceptions of resident connectivity and natural support approaches, as well as the facilitators and barriers to these approaches in urban communities within a Canadian context, utilizing qualitative methodology and methods. To our knowledge, this is the only study that explores *how* to create more naturally supportive connections and environments within communities informally.

## Methods

Qualitative methods were chosen due to limited evidence on perceptions of community natural supports and approaches that facilitate them. Grounded theory methodology enabled the exploration of community-based perceptions regarding resident connectivity and natural support approaches, and developed a theory to capture a comprehensive process for advancing natural supports in urban community settings [26]. The rich, contextualized findings associated with this methodology provided a deeper understanding of experiences and processes within a community [26]. ‘Community’ was defined as the geographical area where residents live. This qualitative study adheres to the Standards for Reporting Qualitative Research.[27] This checklist is included in Supporting Information S1 File.

### Community classification framework

This study aimed to identify a theory encompassing natural supports approaches for communities across varying levels of economic, social and physical vulnerability. To ensure this broad representation, participants were intentionally recruited from communities across all vulnerability classifications.

To guide this effort, this study employed an urban classification framework developed by the City of Calgary Community and Neighbourhood Services, which are municipal representatives responsible for addressing the social needs of local communities and residents.[28] This framework categorized all Calgary, Alberta, Canada communities into three vulnerability subgroups – high, moderate and low –based on differences in economic, social, and physical well-being.[28] Classification was determined using 19 indicators; examples included low-income households and lone-parent families.[28] A complete list of these indicators is provided in Supporting Information S1 Table.

Each indicator was quantified using two complementary metrics. The Index of Volume (INV) captured the *number* of individuals within a community experiencing a specific indicator. The calculated INVs for each indicator within a community were averaged to determine the overall community INV.[28] The Index of Risk (INR) captured the *percentage* of individuals in a community experiencing a particular indicator. Similarly, the INRs for each indicator within a community were averaged to determine the overall community INR [28] Communities were classified into the following vulnerability sub-groups: high INR and high INV (top 40%) were highly vulnerable; those with high INR and low INV, or low INR and high INV were moderately vulnerable; and those with low INR and low INV were the least vulnerable [28].

This framework was used exclusively to guide recruitment and ensure representation across vulnerability classifications, thereby reducing the risk of bias in developing a theory relevant to communities with varied structural and social conditions. Communities from each vulnerability classification (highly, moderately, and least) were randomly selected using randomization software.

### Study sample

Purposive sampling was used to deliberately identify participants who were familiar with the randomly selected community and its available opportunities and activities [29]; these individuals were referred to as *community champions*. The inclusion criteria for community champions included those who 1) resided or worked in the selected communities and were active participants; 2) had lived or worked in their communities for at least six months to ensure familiarity; and 3) could speak English due to the lack of resources for translation. To ensure representation of both formal and informal perspectives within the community, study participants were drawn from multiple groups. Community Social Workers (CSWs) were identified and invited to participate because of their roles in designing, implementing, and evaluating community programs and supports. CSWs contribute to enhancing the health and well-being of communities by collaborating with residents and community-based organizations, initiatives, and partners. The sample also included representatives from Community Associations (CAs). CAs are non-profit, community-based organizations that offer residents social, educational, and recreational opportunities [30]. Residents, mostly comprised those who volunteered or held community leadership roles, were also recruited to provide firsthand accounts of natural supports approaches within their communities.

### Recruitment

Representation across vulnerability classifications was central to this study’s aims; therefore, recruitment was conducted within the communities randomly selected from the City of Calgary classification framework.

A study summary was initially circulated to CSWs by a non-study representative to maintain confidentiality and ensure minimal potential for coercion. CSWs willing to participate connected with the research team directly, and all were included in the study.

After initial purposive sampling among CSWs, theoretical sampling was used to identify additional champion perspectives to include. Theoretical sampling is guided by emerging concepts and categories to clarify connections between themes and categories and to facilitate saturation of categories.(100) After initial interviews with CSWs were conducted and analyzed, theoretical sampling guided expansion to residents in order to gather firsthand experiences of natural support approaches in urban communities. CSWs helped identify other champions to facilitate the collection of rich data. Theoretical sampling also identified CA representatives as a crucial group to connect with to reach our objective. CAs were emailed a summary of the study. Representatives who agreed to participate were then asked to help identify additional champions within their community.

This study aimed to achieve saturation in each community studied and vulnerability classification by connecting with at least three communities in each and at least two champions per community – one a CSW or CA representative and the other a resident to ensure both formal and informal perspectives were gathered. A minimum of three communities per vulnerability classification was proposed to reduce bias and ensure data saturation within study timelines. The recruitment period for this study was March 10, 2019, to August 30, 2019.

### Data collection

**Fig 1:**
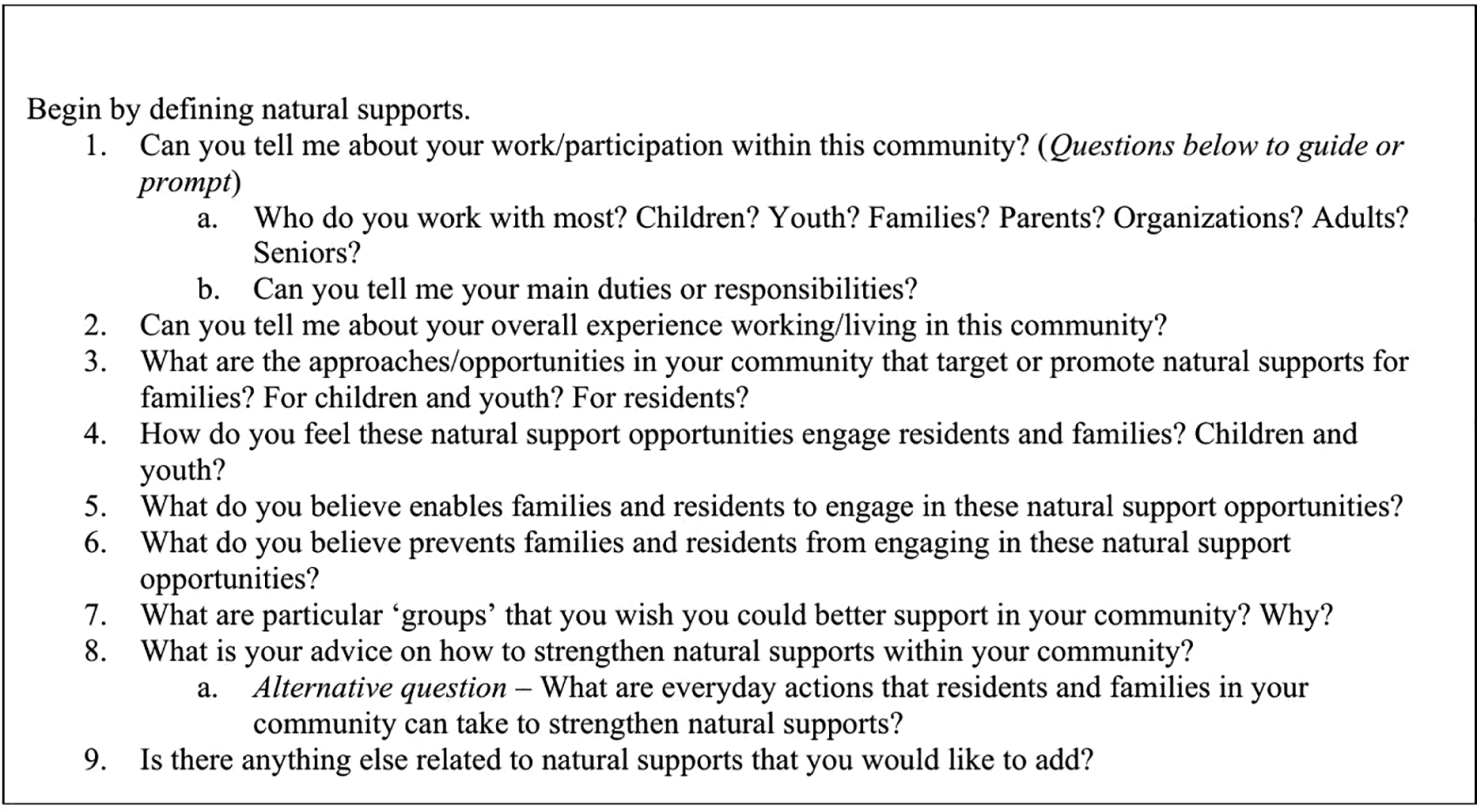
Semi-structured interview questions.

Semi-structured interviews were used to explore perceptions, facilitators, and barriers related to natural supports and approaches that foster them. The semi-structured questions (Fig 1) were designed to align with the study’s objectives and grounded theory methodology [31]. Using cognitive interviews, semi-structured interview questions were pilot-tested with two CSWs, and feedback was obtained to improve the interview guide.

Participants were emailed 48 hours prior to the interview to facilitate preparation. One researcher (JW) conducted the interviews in person or by phone in English, which generally lasted approximately 30–45 minutes. The interviews were audio recorded and transcribed. Community champions from various sectors maximized validity and triangulated results by providing multiple perspectives on natural support approaches, thereby ensuring rich and complex data [32–34]. Demographic information was extracted from the City of Calgary Community Profiles website to describe key indicators for the highly, moderately, and least vulnerable communities included in this study [35].

### Data analysis

Analysis commenced with line-by-line open coding of transcribed interviews using Windows NVivo 12 software [36]. Words and phrases were coded, compared, and grouped into categories based on similar themes and concepts [37]. The iterative process of constant comparison was employed, where codes and emerging categories from new interviews were compared to previous codes and categories [37]. As more interview transcripts were coded and compared, the relationships between categories became evident, leading to the emergence of the core category. Selective coding followed, involving the extraction of data related to categories tied to the core category [37]. This process continued until each category reached theoretical saturation—when collecting more data revealed no new information.[38] Next, theoretical coding integrated and conceptualized the relationships among categories.[39,40] The described analytical processes and memoing facilitated the emergence of a data-driven grounded theory [41]. Measures to enhance methodological rigour and strengthen theoretical sensitivity included ensuring fidelity to champion voices, engaging in memoing and reflexivity journaling, maintaining an audit trail, and holding peer check-ins and team meetings to consider relevance, fit, workability, and potential implications [41–43]. These strategies supported the ongoing development of theoretical sensitivity, which involves conceptualizing codes and categories, remaining open to emergent ideas, iteratively comparing codes and categories, and critically reflecting on the study team’s influence on the analytic process to allow a theory to authentically emerge [40].

### Ethical considerations

This study received ethical approval from the Conjoint Health Research Ethics Board at the University of Calgary (REB18–2003). All champions who expressed interest in participating were included to ensure diversity of sex, ethnicity, sexual orientation, and other identities. We sought informed, written, and voluntary consent from each community champion before the interviews began, including consent for recording the interviews. Minors were not recruited for this study. Champions were informed that interview questions were optional and that they could withdraw their consent for project participation at any time without repercussions. All study team members and transcriptionists signed confidentiality agreements. All data was de-identified and stored on a computer that was encrypted and password-protected. Diverse perspectives from community champions and team members were intentionally integrated into the development of codes, themes, and categories, ensuring that the analysis honoured participant voices, mitigated power imbalances, and aligned with established guidelines for community-based research to support a rich and inclusive interpretation of the data. Finally, champions were contacted for permission to use direct quotations.

## Results

### Participant and community characteristics

Eighteen communities from the Canadian urban center of Calgary were included, out of a total of 212 [44], and 23 champions were interviewed. The community champions comprised seven CSWs, nine CA representatives, and seven residents, with 20 being women and three men. Some CSWs served two communities. Nine communities were classified as highly vulnerable, six as moderately vulnerable, and three as least vulnerable. Moderately vulnerable sub-groups were consolidated due to the recruitment of only two communities in the high INV low INR sub-group. Table 1 outlines the demographics of each vulnerability subgroup, consisting of averages from all communities included in that subgroup.

**Table 1:** Community vulnerability classification sub-group demographics in 2020, with the number of communities recruited for each subgroup (n).

Variable	Vulnerability Classification			
	High (n = 9)	Moderate (n = 6)	Low (n = 3)	Calgary Average
% 0–14 years of age	16.8	14.0	17.7	19
% 65 + years of age	10.9	8.0	7.3	14
% who do not speak English or French	3.0	1.0	1.0	2
% immigrants	31.7	22.5	20.0	31
% newcomers (movers 1 year ago)	20.3	20.3	25.7	16
% identify as a visible minority	38.7	21.8	19.3	36
% identify as Indigenous	5.0	3.8	2.7	3
% lone parent families	21.9	12.2	9.7	14
% living alone	28.0	33.7	27.3	24
% unemployed	11.9	9.0	9.0	9
% renter households	46.8	39.7	28.0	29
% spending >30% of income on shelter	28.7	23.7	17.0	22
% in low income	15.3	10.8	5.7	9
Median household income (CAN$)	69,567.44	96,174.50	124,863.33	97,329
% post-secondary certificate, diploma or degree	48.1	68.7	71.0	61

### Theory for natural supports and their approaches in urban communities

**Fig 2:**
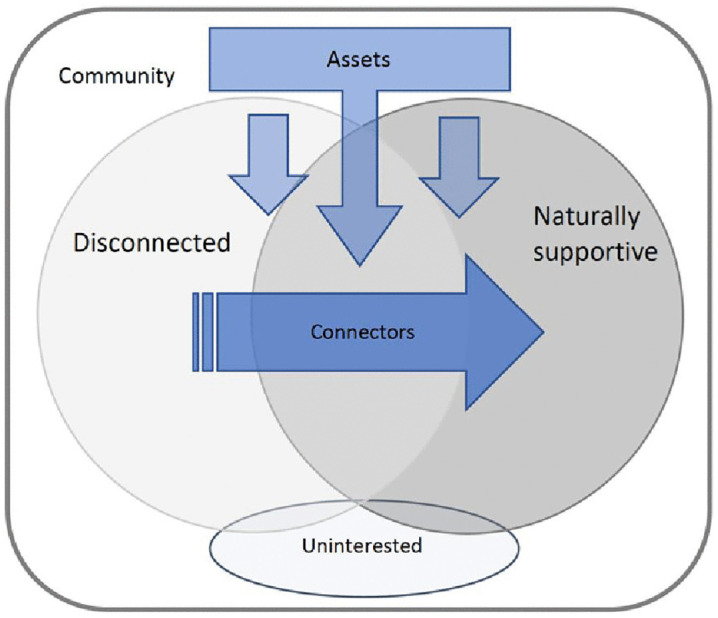
A model of the grounded theory for building social connections using natural supports approaches in communities in a Canadian city.

The grounded theory for natural support approaches in urban communities is: *Building a community’s ability to shift from disconnected to naturally supportive: the need for connectors, assets, and action to empower residents.* Fig 2 illustrates this theory. The theory and figure demonstrate that natural support approaches can transform a disconnected community, where residents feel unable to establish social connections, into a naturally supportive one. Community connectors were essential facilitators for this action and natural support approaches. The availability of community assets further facilitated connectors and natural support approaches. Finally, some residents in each community were uninterested in connecting with others in their community.

Analysis identified six categories, as shown in Table 2. The categories and their related themes are discussed in further detail below, along with supporting champion quotes for each theme in Tables 3, 4.

**Table 2:** Elements of natural supports approaches in urban communities: categories, themes and overview.

Categories	Themes	Overview
***Resident Connectivity*** Disconnected	Fear	Fear was a key factor that prevented residents from developing and participating in natural supports in the community. These included perceptions of the community being unsafe and residents wanting to avoid feeling vulnerable to judgment.
	Isolation	“Silent members” of the community were more likely to experience isolation or were unaware of how to connect with resources and other residents.
Naturally supportive	Sense of community	Residents felt a sense of belonging to their community and “looked out for each other.” Residents also perceived their community as safer.
Uninterested	Uninterested in community connections	Residents were uninterested in connecting with their community. Busy schedules or trying to meet their needs contributed to a lack of participation.
Action	Action was necessary	Actions were used to describe natural supports approaches and the formation of social connections.
***Facilitators & Barriers*** Community connectors*	Making connections	This role needed to make connections and build trust between residents, organizations, schools, etc.
	Input from residents	This role needed to meet and receive input from residents.
	Connecting residents	This role needed to connect residents to supports, services and resources.
	Building community abilities	This role needed to build a community’s problem-solving ability and support and empower residents.
Community assets	Accessibility	Minimizing barriers for residents.
	Inclusivity	Opportunities were open to everyone.
	Partnerships	Promotion and strengthening of partnerships.
	Leadership	Community leadership was accessible, receptive, open to innovation, and involved with residents.
	Availability of resources	The availability of financial, space, childcare, volunteer and staff resources in each community.
*core category		

**Table 3:** Quotes of champion perceptions of *resident connectivity* in urban communities by categories and themes.

Categories and themes	Quotes
***Disconnected***	
Fear	“I think people are cautious about meeting new people and making new connections, and it’s a vulnerable thing to put yourself in a space with people you don’t know and try and get to know them.” [Champion 15]
	“… we do have the refugee families...I would really like to see them more integrated into the community…I think that the language barrier can be, maybe, a fear for them….” [Champion 4]
Isolation	“I mean, they’re part of this community, but … it’s ‘silent’ members of the community… So, most people don’t know where they live or what their issues are, and they don’t participate much in life in the community.” [Champion 11]
	“… they’re more transient. They’re kind of in and out, so they maybe don’t even hang around long enough to access the services, or they’re very isolated and they don’t know. They don’t have connections, they don’t know. No one tells them.” [Champion 3]
***Naturally supportive***	
Sense of community	“But you tip the balance in the equation by having the more connections, the more belonging, the more safety, that my kids can know within their two and a half blocks to school, a dozen houses that they would know are safe, safe for them to go to… And they have a personal connection, too.” [Champion 20]
	“…it was very nice to see that some Indigenous families that had been disconnected from their culture, and this was, for them, kind of a first connection back to introduce the young kids, and to, ‘Oh, this is my culture. This is what we do.’ And you could tell… in people’s reactions and faces, the pride that they had in this event taking place and them being participants in the event.” [Champion 11]
***Uninterested***	
Uninterested in community connections	“I think the neighbourhood is quite opaque for a lot of people…it’s just the place they sleep, and they get everything else that they think they need outside of it.” [Champion 22]
	“Some people will opt out. Some people don’t want to know their neighbour…we can’t make this blanket assumption that everyone wants to be friends with their neighbours or hang out with their neighbours or do anything with their neighbours.” [Champion 3]
***Action***	
Action was necessary	“…it’s kind of a perfect example of how we try to help community members connect with one another, give them more face time with one another. It’s a regular opportunity for residents to meet with one another.” [Champion 2]
	“I think that in order to get to know and build bridges for essentially what are strangers, the more frequent you can see them, the more opportunity there is for that relationship to go deeper.” [Champion 20]

**Table 4:** Quotes of champion perceptions of facilitators and barriers of natural supports approaches in urban communities by categories and themes.

Categories and themes	Quotes
***Community connectors***	
Making connections	“I think that’s the biggest thing, is building a relationship so that they can feel safe with you, and from there, then you can support them. If you don’t have a relationship, you can’t support them.” [Champion 12] “It’s important for kids to feel like they belong in the community…Well, it empowers them. They feel involved.” [Champion 4]
Input from residents	“Yeah, I think the biggest thing is we need to communicate with all people.” [Champion 6] “And so, our board of directors and our residents were really visionary, and a few years ago, they said, we don’t want to put our blood, sweat, and tears into a building just to keep the lights on to rent out to weddings, right? We really want to make sure that we’re a community organization with community impact.” [Champion 23]
Connecting residents	“So, I’ve supported them through the process… connected them with other people within the city… finding the land and getting the permits needed… and just getting organized… and skill building, and it was a lot of coaching….” [Champion 11] “It might help [kids] respect the communities too, because they’ve been engaged in it and they see that people are willing to kind of work with them - you know, because sometimes kids… they kind of get into stuff that isn’t always so great, but if they were to come to some community kind of thing and be engaged, then maybe they’d just respect the community and the people a bit more.” [Champion 5]
Building community abilities	“…I think anything that can be done to help a volunteer, any time somebody takes a leap of faith and risks and says, ‘I want to give this gift to the neighbourhood,’ like, it’s an opportunity to learn and grow and build capacity and connect them and make them a lifelong champion of that neighbourhood....” [Champion 22] “I definitely need builders. I need them to want to do something, and I need them to work with me within the right channels, and then resource them as much as I can… And it’s working… I’m like, ‘Okay, maybe you and I could work and fill out this grant application…’ And so, I’ve got a couple people who have done that and they’re like, ‘Oh, that wasn’t so hard. Look what we managed to do.’” [Champion 14]
***Community assets***	
Accessibility	“I think… trying to reduce barriers… making [opportunities] free is really important, and a lot of times too, if they are kind of close by can help.” [Champion 1] “…we find it’s easier to get families to come out if you’re offering [opportunities] at a certain time of day, if there are supports provided. So, are we feeding them? Do we have childminding? Are they going to get something out of it? So, a lot of our families in the community are busy. They’re struggling. They can’t necessarily always come out...” [Champion 8]
Inclusivity	“And just said… ‘would you like to share about your culture,’ which they were from Pakistan, and the mother, who volunteers regularly with me, but… she’s not confident in her English… she… would never go to a parent-teacher interview, and that is a huge barrier, it’s that piece around having to go and try to communicate in a language that you’re not comfortable in. But she was just so excited about the opportunity to share about Pakistan….” [Champion 10] “…we are going to start a project where we have some seniors… come to that afterschool program. And we’re still kind of in the design phase of it, but really the whole outcome of it is to get the seniors and the kids kind of face-to-face with one another, and… for the kids [to know] seniors who live right down the road from them...” [Champion 2]
Partnerships	“And what I personally have initiated… is actually reached out to our neighbouring Community Associations… and we’ve formed… the [X] Community Network. So [the communities] all came together and said, how can we learn from each other and kind of support each other?” [Champion 18] “…one of the things I have tried to do is link [youth] into other opportunities. So, for instance, the Community Association takes volunteers on for different events. And their volunteers, they connect with the local high school.” [Champion 1]
Leadership	“I think this is one of the projects that we focus on trying to build capacity in the neighbourhood and empower people to take the lead and taking action as well.” [Champion 9] “So, we are trying to identify people in the community that have some potential to take on leadership roles, or some natural abilities, and support them to move into those roles... I think it’s important to have people who are experts in their community... it adds some credibility, it builds leadership, it creates a sense of familiarity among people who attend events.” [Champion 2]
Availability of resources	“The other thing that I think is really important is the use of common spaces, so whether that’s playgroup at the community centre, because people are, I think, reluctant to invite essentially strangers into their home… ‘Come for a play date’ feels like a bit of a huge boundary to leap if you’ve literally just interacted with somebody at the park for 20 minutes. But… it helps if there’s a common place to go in your neighbourhood, whether it’s the Parent Link Centre or the community centre…”. [Champion 20] “We lack volunteers to come up with the ideas that will generate the activity. So, that’s our biggest [barrier]… So, I don’t know… If I knew the answer to that question, I’d try and solve it in terms of our communications.” [Champion 16]

### Perceptions of Resident Connectivity

#### Disconnected.

Community champion perceptions of resident connectivity primarily focused on why residents may or may not have participated in their community (Tables 2, 3). Champions recognized that some residents felt separate from others, leading to perceptions of disconnection. Two main themes emerged from the data in this disconnected category: *fear* and *isolation*. *Fear* indicated a lack of confidence to engage with community members, especially among immigrants and newcomers, due to language and cultural barriers. Additionally, perceptions of community safety contributed to this fear. For instance, champions noted that some residents restricted their children to organized activities and sports, limiting their movement throughout the community because of perceived elevated crime levels. Furthermore, champions perceived that residents facing struggles often wished to keep their circumstances private, fearing vulnerability to judgment and feeling like an inconvenience to their neighbours. As a result, these residents rarely engaged with their community or connected with others.

#### Isolation.

Isolation pertains to “silent members” or specific populations that are more susceptible to experiencing isolation, such as seniors, youth, and at-risk families (defined by champions as those in crisis, with low income, lacking support, or single parents). Experiences of marginalization and misconceptions have contributed to the isolation of some residents. Additionally, social anxieties, especially among youth, have led to a reluctance to engage and connect with other residents. Those who are uncertain about connecting with others and accessing available resources are also more likely to experience isolation in urban communities. Champions identified newcomer, transient, immigrant, and refugee populations as more likely to face challenges in accessing community resources.

#### Uninterested.

Another category consisted of those who were *uninterested in community connections* (Tables 2, 3). The most frequently suggested reasons for the lack of connection from these residents included busy schedules, a focus on meeting their own needs, and a disinterest in community-based opportunities. Residents may also have had different definitions of community and primarily formed connections outside of their residential community; examples included cultural, faith, and work communities. Champions were unable to determine or estimate the proportion of residents in this group and expressed interest in learning more about them in the future. Therefore, the representation of this category in Fig 2 may not accurately reflect the true proportion of residents uninterested in participating in their community.

#### Naturally supportive.

Champion descriptions of the characteristics and benefits of naturally supportive communities emerged as a critical category (Tables 2, 3). A s*ense of community* was a prominent theme, where residents felt a sense of belonging and valued their role as community members. Feeling “at home” in their community fostered more connections with other residents and led to greater involvement in opportunities and events. Residents were also more inclined to “look out for each other.” For example, one resident called a neighbour because his garage door had been left open. Additionally, residents who were comfortable with their neighbours felt safer and more secure in their community. When people felt safe, they were more likely to participate in community-based programming and events, and allow their children and youth to walk to school.

#### Action.

All champions associated community-based natural supports with action (Tables 2, 3). Words such as “bridging,” “building,” “coming together “, “forming,” “making,” and “reaching” were used in conjunction with natural supports, emphasizing the need for action to create community-based connections and relationships. One of the most frequent recommendations made by champions to promote the development of naturally supportive communities was for residents to say “hello” to each other when walking in their community and to know the names of their neighbours. Residents were also more likely to create connections and participate in the community if they could relate to one another by sharing something in common, such as faith, culture, children, and interests. Champions further emphasized the need for in-person opportunities to foster natural supports.

### Facilitators and barriers to community-based natural supports approaches

#### Community connectors.

Every champion emphasized the importance of having a community connector to facilitate urban, community-based natural supports (Tables 2, 4). These connectors were essential for fostering a naturally supportive community and creating connections among residents. Consequently, these connectors became the core category. Terms used to describe connectors included “builders”, “initiators,” “brokers,” “ambassadors,” “block/neighbourhood connectors,” and “engagement coordinators.” The nature of their roles justified the title of these representatives, thereby establishing the core category title. The key themes in this category comprised the primary roles of connectors: 1) *making connections* and building trust with residents, organizations, sponsors, schools, etc.; 2) meeting and gathering *input from residents*; 3) *connecting residents* to supports, services, and resources; and 4) enhanc*ing a community’s capacity* to problem-solve and to support and empower residents and families.

#### Community assets.

Champions perceived that community assets facilitated natural supports and approaches that fostered them (Tables 2, 4). Community assets encompass resources and attributes of both residents and the community. The absence of an asset was often viewed as a barrier. There were five key themes in the community assets category, each described by its corresponding asset type. The first asset type was *accessibility*, which included strategies to reduce barriers for residents, enabling the creation of natural supports. This involved identifying nearby locations for activities and events (e.g., community centres or local schools), providing free or low-cost opportunities and equipment, and helping families meet basic needs through food assistance and subsidies. Champions also emphasized the need for increased awareness of opportunities by utilizing various advertising modalities (e.g., door-knocking, flyers, social media, and emails). However, champions noted that the most effective way to promote opportunities and connections was for residents to invite each other, as they tend to trust fellow residents more.

The second asset type was *inclusivity*. While some approaches may target specific populations, champions recommended that the majority should be accessible to everyone so that whole families and individual residents can attend. Opportunities for residents to share their culture, traditions, customs, and language help minimize cultural and language barriers. Approaches promoting intergenerational opportunities were considered a potential “untapped” resource in communities; examples include “adopt” a grandparent or grandchild, after-school mentoring and homework assistance, and cooking classes.

The third asset type was the promotion and enhancement of *partnerships*. The three most common partners suggested for communities were local schools, community organizations and non-profits, and neighbouring communities and CAs. Collaborating with these community-based entities facilitated greater awareness of opportunities and strategies, as well as the sharing of knowledge and available resources.

The fourth type is related to essential *leadership* qualities that foster natural supports. Characteristics of accessible and receptive community leadership included active participation within the community, openness to new ideas and innovations, empowering residents, and assisting residents and connectors in overcoming potential obstacles (such as bylaws or other legalities) to natural supports approaches. Additionally, champions emphasized the importance of involving residents, including children and youth, in planning activities and approaches. To promote sustainability, ideally, some opportunities were resident-led. Lastly, champions emphasized the need to update or redefine the role of CAs within communities. They provided recommendations for CAs to prioritize community engagement to help address the needs of their residents and families.

The fifth asset type involved identifying *resources* to facilitate natural support approaches in urban communities. *Financial* resources for communities included funding for opportunities and equipment, as well as access to neighbourhood grants or monetary aid. Champions explicitly highlighted the availability of funding for community connectors to foster natural supports. The resource of *space* refers to the accessibility of community areas that allow residents and families to connect, encompassing drop-in centers and free venues. Outdoor green spaces offered opportunities to promote strategies for outdoor physical activities and games. However, champions often noted barriers related to access to space, particularly in indoor areas of buildings owned by organizations, associations, schools, and businesses, due to legal issues, costs, liability concerns, and a lack of awareness. *Childcare* was another essential resource; champions recommended providing supervised play areas for children during community events to enable more families to participate. *Volunteers* were crucial to community efforts. Many communities struggled to recruit volunteers and sought strategies to enhance volunteer numbers. Champions noted that residents were reluctant to volunteer, particularly in child and youth initiatives, due to training requirements, police checks, and concerns about liability. The few communities with sufficient volunteers highlighted that making volunteering enjoyable, offering support and training, providing various volunteer opportunities, and frequently expressing appreciation were vital for recruitment and retention. Lastly, *staffing* referred to the availability of organizers and coordinators who were familiar to residents and could build trust. Champions recommended that approaches targeting vulnerable children, youth, and families include staff with proper training to connect with this specific population and direct them to available resources. Staff with these skill sets were viewed as essential in developing naturally supportive communities, especially among those who are more vulnerable.

## Discussion

Natural supports approaches are key mechanisms for communities to empower and promote the well-being of residents and families. As shown in the data-driven theoretical model (Fig 2), champions identified that community connectors, assets and actions were necessary to shift communities from disconnected to naturally supportive. To illustrate this theory, the promotion of natural supports approaches requires communities to identify connectors (i.e., those whose responsibilities or roles include making connections, receiving input and building a community’s capacity), such as CSWs, CAs, block leaders, café owners, and volunteers. Communities then need to take stock of community assets to mitigate barriers for offering natural supports approaches, and to enable residents and families to participate in these approaches by improving accessibility (e.g., providing transportation, making events free entry), being inclusive (e.g., events that are open to everyone of every age, ethnicity, English proficiency, sexual orientation, etc.), and optimizing available resources (e.g., community planning to ensure large enough green spaces, and supporting community volunteers and staff). Accessible, respectful, and responsive leadership, as well as developing partnerships with community organizations and businesses, for example, are also key assets to consider that help facilitate natural support approaches and decrease barriers for residents and families to engage in these approaches. The remainder of this discussion presents key themes and their alignment with existing literature, followed by the implications of this study for communities, community planners, and policy.

### Perceptions of resident connectivity

This study revealed that some residents feared social connections or felt isolated and, as a result, were perceived as disconnected from others in their community. These findings were predominantly related to perceptions of community safety. Despite decreasing crime statistics in Canadian national reports over recent decades [45], many residents were perceived to view their communities as unsafe. A lack of safety can cause residents to minimize interactions with others in the community and become less likely to intervene or offer help when problems arise [46]. Providing opportunities for residents and families to engage with diverse people and places in their community can foster a greater sense of belonging and security [47].

Residents may be less likely to engage with their community due to a lack of interest in connecting with neighbours or having friends or partners living outside the community [48]. Residents may also struggle to find time to participate. Twenty-four percent of men and 38% of women in dual-income families in Canada experience severe ‘time-crunch’ stress [49], which may lead many families to feel overwhelmed and focused on meeting their own needs. Another possible explanation is that families seek resources outside their communities due to limited recreational and cultural activities, as well as other available resources [50]. It is important to note that while individuals may be uninterested in residential community connections, this does not necessarily imply a lack of natural supports. Future research should aim to identify the proportion of residents in this group within urban communities and explore effective strategies to engage them, particularly if they are new to the community or are experiencing isolation.

A sense of community emerged as a key theme in naturally supportive communities. Despite varying definitions of a sense of community in literature, there is a consensus that emotional interconnectedness among residents of a healthy community promotes well-being for individuals, families and the community [48,51–53]. The social-ecological model and social capital theories support this idea [54,55]. Brief, repeated interactions with neighbours can foster a greater sense of belonging, and over time, trust and familiarity can develop. In line with the broaden-and-build theory, a stronger sense of community and belonging enhances resident well-being [56]. Residents with optimal well-being are more likely to engage with others and participate in their community [56]. These actions create an effect where positive interactions and social connections boost overall psychological and physical well-being, encouraging further engagement in community, work, and school environments [57]. Such approaches may also yield significant social returns on investment for individual residents and the communities with which they engage.

### Facilitators and barriers to natural supports approaches

Community connectors are essential for fostering community-based natural supports. Most peer-reviewed literature on connectors pertains to health services. For instance, a study of rural health services described community connectors as mechanisms for reaching groups that are often difficult for service providers to access, such as low-income families, seniors, and youth [58]. Social prescription is another example where healthcare providers can collaborate with a community link worker, whose responsibilities include connecting patients with community-based programs and resources [20]. However, these interventions necessitate access to health professionals or awareness of other formal community resources. Similar to this study, some literature identifies community connectors as key drivers for enabling informal community strategies and social connections [59,60]. The findings from this study enhance existing work by underscoring the contemporary significance of community connectors in developing and strengthening a community’s well-being through informal natural supports and approaches that foster them.

Community assets enhance a community’s ability to foster natural supports while empowering community connectors, residents, and families. Although each community possesses contextualized assets, a core category and five key themes were identified that promote informal natural support approaches. The literature highlights many of these facilitators, particularly within community asset mapping [61,62]. Asset mapping identifies and builds upon what communities currently have, promoting a strengths-based approach to developing community-based strategies [61,62]. This study distinguishes itself from other asset mapping studies by identifying specific assets that support natural support approaches in community settings. Researchers involved in community asset mapping projects, especially those evaluating them, have found that approaches and strategies that leverage and build on the community’s assets are more likely to be effective than introducing external assets [63].

The remainder of this discussion focuses on novel community asset findings and key contemporary barriers identified by community champions. Champions recommended approaches that leverage these assets and offered suggestions to address the identified barriers, noting that such strategies can be feasible within limited community resources through the restructuring, re-evaluating, and redistributing of community priority areas and resources. The first barrier to natural supports approaches is the CA’s contemporary role and future. CAs often face significant challenges due to continual decreases in available funding and issues with facility maintenance [64]. They are also subject to numerous priorities and must continually adapt to changing community dynamics and needs [65]. Literature has identified that CAs are vital assets to communities [66,67]. Furthermore, collaboration between CAs and residents, organizations, businesses, schools, and other entities empowers a community’s residents and leadership, optimizing resources to achieve innovative and sustainable goals [60]. One goal is to promote community well-being while also targeting and supporting those who are vulnerable. This study emphasizes the importance of CAs for engagement and facilitation of natural supports for residents, highlighting the need for CAs to prioritize natural support approaches that address the needs and goals of residents, while also recognizing the competing demands for amenity and facility maintenance, as well as revenue. CAs can be key facilitators of natural support approaches by engaging with community connectors and identifying community assets.

Access to both indoor and outdoor spaces facilitated natural support approaches. Specifically, champions highlighted the need for drop-in and free community spaces, aligning with findings in peer-reviewed literature [68–70]. However, champions frequently mentioned restricted access, legal concerns, and potential liabilities as significant barriers to community-based spaces, such as the lack of available kitchen space in community centers for local events. Other access challenges included making rent payments, adhering to school restrictions, and having limited green space for outdoor activities. Community leadership, along with local schools, organizations, and businesses, should consider making their policies and procedures more adaptable to natural support approaches for families and residents.

Many champions expressed concerns about recruiting and retaining volunteers. Volunteers are a crucial resource for community initiatives and events. However, significant barriers to volunteering exist, especially for child and youth strategies, due to training requirements, police security checks, and potential liabilities. According to a 2010 report, the largest barrier to volunteering for Canadians was a lack of time [71]. Interestingly, 45% of non-volunteers had never volunteered because they had not been asked and were unaware of available opportunities [71]. Nesbit *et al.* conducted a comprehensive review of the volunteering literature [72]. They developed a conceptual framework with suggestions on how organizational characteristics, volunteer management, and environmental factors can influence volunteer involvement [72]. One example includes recommendations for communities and organizations to appoint a dedicated, full-time, paid volunteer manager [72].

### Potential limitations

This study has two potential limitations. The first pertains to only six communities that recruited resident or volunteer champions for the study. However, at least one resident or volunteer was interviewed within each vulnerability classification subgroup, ensuring data collection from these perspectives. Secondly, due to the inherent biases associated with opt-in recruitment and the practice of having community champions nominate other champions, there may be reduced heterogeneity among the participants in this study relative to the broader community population that would be captured through more randomized or stratified recruitment. Individuals with similar values and interests may be more likely to respond to recruitment emails and nominate others who share these values and interests. Community connectors likely constituted a significant portion of the champions in this study. However, connectors are some of the most knowledgeable about resident connectivity, assets, barriers, and opportunities in communities, making them essential individuals to interview for this study. Given the critical role of connectors in fostering natural supports in communities, the authors are confident that the core category would have emerged as community connectors, regardless of the participant composition.

### Implications

To our knowledge, this study is the first to explore urban community-based informal natural supports and approaches that foster them from the perspectives of community champions. The theory and, particularly, asset themes identified in this study can help communities develop natural supports and create opportunities for residents and families to build supportive community environments. Community champions shared their views on the benefits of naturally supportive communities, particularly regarding the enhanced sense of safety, security, and belonging that residents experience. Natural support approaches offer an innovative and sustainable opportunity for communities to enhance their residents’ well-being by leveraging existing assets and resources to reduce the impact of barriers. As noted by the community champions, actions as simple as saying “hello” to others while walking or learning neighbours’ names contribute to building naturally supportive communities and enhancing resident well-being.

Communities are uniquely positioned to identify the needs of their residents and provide contextualized support to address these needs [73]. A healthy and empowered community is more likely to problem-solve and create innovative, sustainable solutions to community or societal issues. A relevant and recent example is the COVID-19 pandemic. Evidence from the pandemic indicates that mandated social isolation and lockdowns increased the risk of poor mental health, loneliness, and stress in individuals globally [74–77]. Furthermore, children and youth may be more vulnerable than other age groups to negative long-term outcomes from the critical periods of brain and social-emotional development that coincided with physical distancing, social isolation, and the closure of schools and recreational spaces [78]. Many societies are still grappling with the repercussions of the pandemic and its impact on mental health and well-being. Awareness of the significance of natural supports, community, neighbours, and green spaces grew during the pandemic [79,80]. Implementing natural support approaches in communities to promote resident connectivity and well-being has never been timelier. This study offers a theoretical framework for *how* communities can foster informal natural supports and their approaches. Evaluating natural support methods and the theory presented in this study are important next steps for future research.

### Potential action points

Although this paper introduces many potential action points, our findings have identified six crucial ones for overcoming frequent barriers to natural support approaches. First, the asset themes uncovered in this study can help communities develop natural supports, specifically. However, general knowledge of these assets and how to access them may be limited within communities [81]. Identifying relevant community assets specific to natural supports and raising awareness among residents are essential for implementing these strategies.

Next, community-based leadership, businesses, organizations, schools, associations and other entities should recognize the value of the community connector role and leverage support and resources for this role to promote natural supports approaches. Once appropriate resources have been allocated, identifying community connectors — such as individuals who already hold these roles and responsibilities or those who can be trained — is a critical step for natural supports, as connectors are key facilitators.

Third, CAs and other community-based organizations aiming to enhance natural supports and supportive communities should consider reviewing their priority areas and incorporating natural supports approaches into local planning and evaluation. This process can include creating or referencing indicators [82] for natural supports approaches, which are essential for effective community planning and evaluation.

Fourth, partnerships with local organizations, businesses, associations, funders, and surrounding communities will leverage knowledge and resources to foster natural supports among residents. Exploring potential partnerships is a crucial step in identifying community assets, such as collaborating with organizations that offer community grants or other forms of support.

Fifth, policies and procedures, including bylaws that address access to community spaces (e.g., free spaces, community buildings, unused business spaces), would benefit from a comprehensive review to manage risk and enhance usage, particularly in implementing natural supports approaches.

Ultimately, discussing the benefits and opportunities of volunteering may enhance the recruitment and retention of volunteers. Effective volunteer coordination and recognition are also essential, as suggested by community champions in this study and literature [72].

## Conclusion

In summary, community champions identified that community connectors, assets, and actions were needed to transform communities from disconnected to naturally supportive. Recognizing community assets and supporting community connectors were pivotal to informal natural support approaches, which foster resident connectivity and well-being in urban communities. With the information gathered from this study, knowledge users, such as community planners and policymakers, will gain a better understanding of how to optimally invest in natural support approaches and develop community approaches that enhance natural supports and the well-being of their residents and families.

## Supporting information

S1 File Standard for Reporting Qualitative Research checklist. (DOCX)

S1 Table Indicators used for community vulnerability classification, from the City of Calgary Indices of Community Well-being report. (DOCX)
