# Supplementary material for: Community perceptions of natural supports and their approaches: A Canadian grounded theory study with Connections First
Source: PLoS One. 2026 May 4;21(5):e0346971. doi: 10.1371/journal.pone.0346971 (PMC13138642; doi:10.1371/journal.pone.0346971)
Supplement: S1 Table — (DOCX) [file pone.0346971.s002.docx]

**S1 Table**. Indicators used for community vulnerability classification, from the City of Calgary Indices of Community Well-being report.

| **Dimension** | **Sub-dimension** | **Indicators** |
| --- | --- | --- |
| **Economic well-being** | Poverty | Persons in low-income households |
|  |  | Children in households receiving provincial income support |
|  |  | Seniors receiving Guaranteed Income Supplement |
|  | Employment | Unemployed adults |
|  |  | Unemployed youth |
|  | Housing | Renters spending >30% of income on shelter costs |
|  |  | Dwellings requiring major repair |
| **Social well-being** | Family stability | Lone-parent families |
|  | Social inclusion | Recent movers |
|  |  | Recent immigrants |
|  |  | Official language ability |
|  |  | Unattached individuals |
|  |  | Seniors living alone |
|  | Education | Persons not completing high school |
| **Physical well-being** | Personal health | Hospital in-patients |
|  |  | Persons with disabilities |
|  | Personal safety | Emergency room visits |
|  |  | Person crimes |
|  |  | Residential structure fires |
